# Insulin-like growth factor 1 receptor expression and *IGF1R* 3129G > T polymorphism are associated with response to neoadjuvant chemotherapy in breast cancer patients: results from the NEOZOTAC trial (BOOG 2010-01)

**DOI:** 10.1186/s13058-015-0663-3

**Published:** 2016-01-06

**Authors:** Stefanie de Groot, Ayoub Charehbili, Hanneke W. M. van Laarhoven, Antien L. Mooyaart, N. Geeske Dekker-Ensink, Saskia van de Ven, Laura G. M. Janssen, Jesse J. Swen, Vincent T. H. B. M. Smit, Joan B. Heijns, Lonneke W. Kessels, Tahar van der Straaten, Stefan Böhringer, Hans Gelderblom, Jacobus J. M. van der Hoeven, Henk-Jan Guchelaar, Hanno Pijl, Judith R. Kroep, on behalf of the Dutch Breast Cancer Research Group

**Affiliations:** Department of Medical Oncology, Leiden University Medical Center, Albinusdreef 2, P.O. Box 9600, 2300 RC Leiden, The Netherlands; Department of Surgery, Leiden University Medical Center, Albinusdreef 2, P.O. Box 9600, 2300 RC Leiden, The Netherlands; Department of Medical Oncology, Academic Medical Center, Meibergdreef 9, P.O. Box 22660, 1100 DD Amsterdam, The Netherlands; Department of Pathology, Leiden University Medical Center, Albinusdreef 2, P.O. Box 9600, 2300 RC Leiden, The Netherlands; Department of Radiotherapy, University Medical Center Utrecht, Heidelberglaan 100, P.O. Box 85500, 3508 GA Utrecht, The Netherlands; Department of Endocrinology, Leiden University Medical Center, Albinusdreef 2, P.O. Box 9600, 2300 RC Leiden, The Netherlands; Department of Clinical Pharmacy and Toxicology, Leiden University Medical Center, Albinusdreef 2, P.O. Box 9600, 2300 RC Leiden, The Netherlands; Department of Medical Oncology, Amphia Hospital, Langendijk 75, P.O. Box 90157, 4800 RL Breda, The Netherlands; Department of Medical Oncology, Deventer Hospital, Nico Bolkesteinlaan 75, P.O. Box 5001, 7400 GC Deventer, The Netherlands; Department of Medical Statistics and Bioinformatics, Leiden University Medical Center, Albinusdreef 2, P.O. Box 9600, 2300 RC Leiden, The Netherlands

**Keywords:** Breast cancer, Neoadjuvant chemotherapy, Glucose, Insulin, IGF-1, IGF-BP3, IGF-1R, Miller and Payne, Pathological complete response, Single nucleotide polymorphisms

## Abstract

**Background:**

The insulin-like growth factor 1 (IGF-1) pathway is involved in cell growth and proliferation and is associated with tumorigenesis and therapy resistance. This study aims to elucidate whether variation in the IGF-1 pathway is predictive for pathologic response in early HER2 negative breast cancer (BC) patients, taking part in the phase III NEOZOTAC trial, randomizing between 6 cycles of neoadjuvant TAC chemotherapy with or without zoledronic acid.

**Methods:**

Formalin-fixed paraffin-embedded tissue samples of pre-chemotherapy biopsies and operation specimens were collected for analysis of IGF-1 receptor (IGF-1R) expression (n = 216) and for analysis of 8 candidate single nucleotide polymorphisms (SNPs) in genes of the IGF-1 pathway (n = 184) using OpenArray® RealTime PCR. Associations with patient and tumor characteristics and chemotherapy response according to Miller and Payne pathologic response were performed using chi-square and regression analysis.

**Results:**

During chemotherapy, a significant number of tumors (47.2 %) showed a decrease in IGF-1R expression, while in a small number of tumors an upregulation was seen (15.1 %). IGF-1R expression before treatment was not associated with pathological response, however, absence of IGF-1R expression after treatment was associated with a better response in multivariate analysis (*P = 0.006*) and patients with a decrease in expression during treatment showed a better response to chemotherapy as well (*P = 0.020*)*.* Moreover, the variant T allele of 3129G > T in *IGF1R* (rs2016347) was associated with a better pathological response in multivariate analysis (*P = 0.032*).

**Conclusions:**

Absent or diminished expression of IGF-1R after neoadjuvant chemotherapy was associated with a better pathological response. Additionally, we found a SNP (rs2016347) in *IGF1R* as a potential predictive marker for chemotherapy efficacy in BC patients treated with TAC.

**Trial registration:**

ClinicalTrials.gov NCT01099436. Registered April 6, 2010.

## Background

Insulin-like growth factor (IGF)-1 and other members of the IGF-1 pathway have been associated with development, progression, and metastasis of several cancers [1, 2]. Additionally, epidemiologic studies have shown a relation between high circulating IGF-1 levels, breast density [3], and risk of breast cancer (BC) [4]. Increased IGF-1 levels are associated with an elevated BC mortality [5] and with inherent resistance to anti-tumor treatments in preclinical models [6–9]. Furthermore, the IGF-1 receptor (IGF-1R), a transmembrane tyrosine kinase, is frequently upregulated in BC [10, 11]. The biological activity of IGF-1 and IGF-2 depends on binding with the insulin-like growth factor binding proteins (IGF-BPs), mainly IGF-BP3 [12, 13]. Both IGFs bind the IGF-1R and activate the Ras/mitogen-activated protein kinase (MAPK) and phosphatidylinositol-3-kinase (PI3K)/AKT pathways, through which cell proliferation is stimulated and apoptosis is inhibited, respectively [14, 15]. Additionally, the IGF-1R and the estrogen receptor (ER) have been shown to work synergistically, whereby activated ER binds to the promoter regions of *IGF1R* to promote transcription and IGF-1 is able to activate unliganded ER [16, 17].

Previous research has shown that low IGF-1R expression in the tumor is predictive for pathological complete response (pCR) in ER-positive tumors [10] and that upregulation of IGF-1R during chemotherapy predicts a poor outcome in a relative small, heterogeneous group of BC patients [18]. Moreover, genes encoding members of the IGF-1 pathway are known to harbor several single nucleotide polymorphisms (SNPs) that influence the activity of the pathway. SNPs associated with IGF-1 and IGF-BP3 plasma levels and breast density are described [19, 20] as well as SNPs associated with therapy resistance and outcome [21, 22].

Neoadjuvant chemotherapy has been demonstrated to be equivalent to adjuvant chemotherapy for BC survival. This treatment has the advantage of more frequent breast-conserving therapy [23] and offers the opportunity for translational research of molecular predictors of tumor response. Additionally, the Miller and Payne (MP) histological grading system can be used to assess response to neoadjuvant chemotherapy because it is associated with patients’ disease-free and overall survival [24, 25]. This study evaluates the expression of the IGF-1R of the tumor before and after neoadjuvant chemotherapy and whether it predicts pathological response according to MP classification after neoadjuvant chemotherapy in human epidermal growth factor receptor 2 (HER2)-negative early BC patients treated in the NEOZOTAC trial [26]. Moreover, we aim to identify SNPs, which have been described to influence the activity of the IGF-1 pathway, to predict chemotherapy efficacy in this cohort. In addition, these SNPs are tested for association with the occurrence of side effects.

## Methods

### Study population

From July 2010 until April 2012, 250 women participated in the multicenter phase III NEOZOTAC trial, randomizing between TAC chemotherapy (75 mg/m^2^ docetaxel, 50 mg/m^2^ doxorubicin, and 500 mg/m^2^ cyclophosphamide) with or without zoledronic acid (4 mg within 24 hours after chemotherapy). Eligible patients had a histologically confirmed diagnosis of HER2-negative stage II or III BC. Other inclusion and exclusion criteria have been described elsewhere [26]. Tumor regression was scored according to the MP classification [24]. pCR was defined as the absence of residual invasive cancer within the breast and lymph nodes [24]. Side effects and hematological toxicity were graded according to the Common Terminology Criteria for Adverse Events version 4.0 (CTCAE v.4.0) [27]. All patients gave written informed consent. The study was conducted in accordance with the Declaration of Helsinki (2008) and approved by the Ethics Committee of the Leiden University Medical Center in agreement with the Dutch law for medical research involving humans.

### Immunohistochemistry

**Fig. 1:**
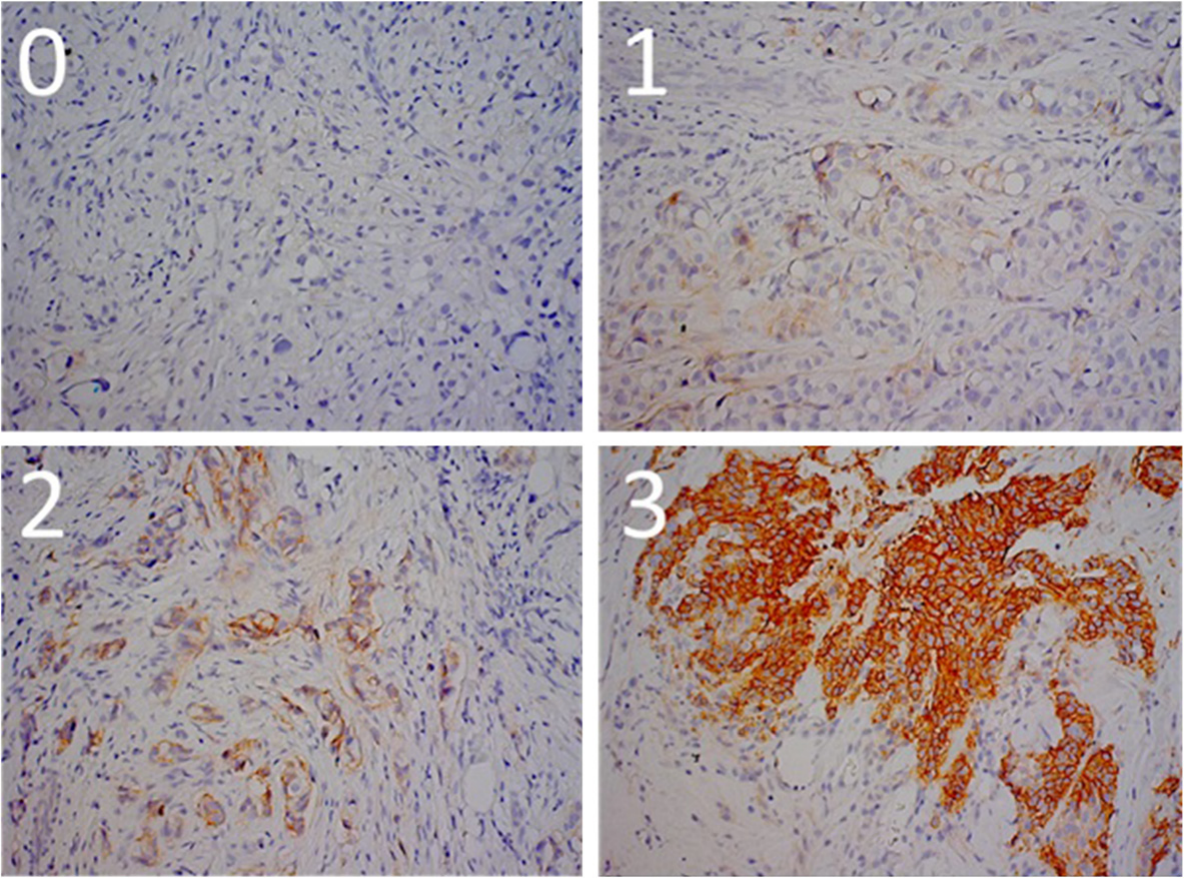
Examples of the membranous IGF-1R staining in breast tumor tissue sections. *Score 0*: staining is observed in <10 % of the tumor cells. *Score 1+*: incomplete staining is observed in >10 % of the tumor cells, *Score 2+*: weak or moderate complete staining is observed in >10 % of the tumor cells, *Score 3+*: strong complete staining is observed >10 % of tumor cells. Samples were considered negative if 0 or 1+ was scored, and positive if 2+ and 3+ was given

Formalin-fixed paraffin-embedded (FFPE) tumor tissue samples of prechemotherapy biopsies and operation specimens were collected for analysis of IGF-1R expression using immunohistochemistry (IHC). From each FFPE tumor tissue sample, one section of 4 μm was cut and deparaffinized with xylene, rehydrated through graded alcohol, and rinsed in distilled water. After blocking of endogenous peroxidase activity with 0.3 % H_2_O_2_ for 20 minutes, heat-induced antigen retrieval was performed in the EnVision™ Flex Target Retrieval Solution in PT Link (Dako, Glostrup, Denmark) at low pH. After blocking with 5 % normal goat serum to reduce aspecific binding by the primary antibody, the sections were incubated overnight at room temperature in a humidified chamber with the IGF-1R antibody (IGF-1 receptor β, D4O6W, rabbit monoclonal; Cell Signaling Technology, Danvers, MA, USA) diluted in phosphate-buffered saline (PBS)/bovine serum albumin (BSA) 1 % at a dilution of 1:200. After the primary antibody incubation, the sections were washed with PBS and incubated with a secondary anti-rabbit antibody EnVision™ (Dako, Glostrup, Denmark) for 30 minutes and visualized using liquid DAB+ (Dako, Glostrup, Denmark). Eventually, sections were counterstained with Mayer’s hematoxylin, dehydrated, and subsequently permanently mounted with Pertex (Histolab, Gothenburg, Sweden). Breast and placenta sections that had previously been identified to express the IGF-1R were used as positive controls, and sections that underwent the IHC staining procedure without application of primary antibodies served as negative controls. Membranous IGF-1R expression was scored on a scale of 0–3+ (see Fig. 1). Samples were considered negative if 0 or 1+ was scored, and positive if 2+ and 3+ was given. The staining was scored by two independent researchers (SdG and ALM).

### SNP selection

To select relevant SNPs in the IGF-1 pathway, a PubMed search with the keywords “IGF-1”, “IGF-2”, “IGF-BP3”, “IGF-1R”, “single nucleotide polymorphism”, “breast cancer”, and/or “clinical outcome” was conducted in July 2013. SNPs that were associated with IGF-1 or IGF-BP3 plasma levels, BC risk, or clinical outcome in cancer patients treated with chemotherapy, were selected. SNPs with a minor allele frequency (MAF) >0.01 in a Caucasian population according to the HapMap project database and with a potential functionality according to the literature review or using national institutes of health functionality database were selected [28]. To minimize the number of tested associations, tagging SNPs were selected for SNPs that were in linkage disequilibrium (*r*^2^ > 0.7). The selected SNPs in the *IGF1*, *IGF2*, *IGFBP3*, and *IGF1R* genes are summarized in Table 1.

**Table 1:** Selected SNPs in IGF-1 pathway

RS number	Gene	Alleles (major > minor)	Position in gene and functionality	Clinical influence of polymorphism
rs10735380	*IGF1*	A > G	Transcription factor binding site, intronic	Variant G allele associated with increased serum IGF-1 level [20, 35, 41]
rs1520220	*IGF1*	C > G	Intronic	Variant G allele associated with increased serum IGF-1 level [35, 42] and BC risk [42].
rs6220	*IGF1*	A > G	3′-untranslated region, microRNA binding site	Variant G allele associated with increased serum IGF-1 level and increased BC risk [42]
rs2946834	*IGF1*	G > A	3′-untranslated region	Variant A allele associated with increased serum IGF-1 level [35, 42] and with worse outcome in BC [21]
rs2270628	*IGFBP3*	C > T	Downstream	Variant T allele associated with decreased serum IGF-BP3 level [20, 35, 36]
rs2854746	*IGFBP3*	G > C	Nonsynonymous in exon 1	Variant C allele associated with increased serum IGF-BP3 level [20, 35, 36, 43] and with better outcome in advanced gastric cancer treated with CT [44]
			(Ala32Gly)	
rs4320932	*IGF2*	T > C	Transcription factor binding site, intronic	Variant C allele associated with worse outcome in ovarian cancer and worse response to CT [45]
rs2016347	*IGF1R*	G > T	3′-untranslated region, microRNA binding site	Variant T allele associated with better outcome in ER+ BC [22]

SNPs selected on basis of literature research and the clinical influence. *rs reference SNP number*, *BC* breast cancer, *CT* chemotherapy, *ER* estrogen receptor, *IGF* insulin-like growth factor, *IGFBP3* insulin-like growth factor binding protein 3, *IGF1R* insulin-like growth factor 1 receptor, *SNP* single nucleotide polymorphism

### DNA isolation and preamplification

DNA was extracted from FFPE tissue samples. Preferentially, tissue from tumor-negative breast tissue and tumor-negative lymph nodes was used (*N* = 95); however, when this was unavailable or unclear from the pathology report tissue from tumor-containing blocks was used. Three sections of 4 μm were incubated overnight at 50 °C in 500 μl lysis buffer (NH_4_Cl 8.4 g/l, KHCO_3_ 1.0 g/l, proteinase K 0.25 mg/ml). Next, 300 μl was taken to extract DNA using the Maxwell forensic DNA isolation kit (Promega, Leiden, the Netherlands) according to the manufacturer’s protocol. DNA isolated from FFPE tissue is cross-linked and fragmented into pieces with a length of a few hundred base pairs. To make DNA isolated from FFPE tissue more suitable for genotyping, preamplification was accomplished for enrichment of the target DNA [29]. The preamplification step consisted of a PCR reaction with eight diluted TaqMan assays (LifeTechnologies, Nieuwerkerk aan den IJssel, the Netherlands) and was performed using the following protocol; to 2.5 μl DNA, 1 μl of a dilution of eight TaqMan assays (pooled at a final concentration of 0.2×) and 3.5 μl HotStarTaq DNA polymerase was added and amplified on a conventional PCR machine. The following PCR conditions were used; 10 minutes at 95 °C followed by 18 cycles each consisting of 15 seconds at 95 °C and 4 minutes at 60 °C. The mixture was diluted 15 times and 2 μl was used for real-time PCR analysis. The selected SNPs were analyzed using TaqMan OpenArray® technology (Life Technologies); however, in case of low call rate, missing samples were reanalyzed separately using the Viia7 RealTime PCR system (Life Technologies).

### Statistical analysis

Possible associations between parameters were analyzed using Pearson’s chi-square test and logistic regression. Univariate and multivariate odds ratios (ORs), 95 % confidence intervals (CIs), and *P* values were derived from logistic regressions. IGF-1R expression and clinical variables, which have been reported to be associated with pCR, were tested in univariate analysis (e.g., hormone receptor (HR) status and clinical T status). The association between IGF-1R expression and MP classification were tested using a logistic ordinal regression where MP classification groups were treated as ordered. In multivariate analyses, parameters were adjusted for covariates with *P* <0.1. We also reanalyzed the latter model using linear regression to check for linearity of relationship between IGF-1R expression and MP classification.

Genotype distributions were tested for adherence to Hardy–Weinberg equilibrium and SNPs significant at the 0.05 level after Bonferroni correction were excluded from the analysis. Genotypes found to be (borderline) significant in the univariate logistic regression models were carried forward to the multivariate model, adjusting for covariates with *P* < 0.1. To correct for multiple testing, a global score test including all SNPs was performed [30]. The score test assumes that the regression coefficients of the SNPs are normally distributed and tests whether the variance of this distribution is bigger than zero. In that case at least one regression coefficient has to be unequal to zero. To investigate the individual, relative contribution of SNPs, a classification and regression tree (CART) was computed (Statistical Package for Social Sciences (SPSS): classify, tree; (IBM Corp., Armonk, NY, USA)). A receiver operating characteristic curve and the area under the curve (AUC) were computed for the predicted probabilities of the CART. The global *P* value was computed using the package *globaltest* in R version 3.1.3 (The R Foundation for Statistical Computing, Institute for Statistics and Mathematics, Vienna, Austria). All other analyses were computed using SPSS software™ 20.0 (IBM Corp.). A significance level of 0.05 was used for all tests.

## Results

### Patient characteristics

Patients of both study arms, chemotherapy with or without zoledronic acid, were included in this study, as no differences were found between both arms regarding pathological response [26]. FFPE tissue was available from 216 (86.4 %) of 250 patients. Clinical characteristics of the 216 patients are presented in Table 2, which are comparable with the characteristics of the entire cohort of the NEOZOTAC trial [26]. Almost 12 % of the patients had a pCR.

**Table 2:** Patient characteristics

	Patients (*N* = 216) in NEOZOTAC	
Median age, years (range)		49.5 (28–70)
Median BMI, kg/m^2^ (range)		26.2 (18.3–42.0)
Clinical T stage	cT1 or cT2	123 (56.9 %)
	cT3 or cT4	93 (43.1 %)
Clinical N stage	cN0	101 (46.8 %)
	cN+	115 (53.2 %)
Tumor type	Ductal	128 (59.3 %)
	Lobular	38 (17.6 %)
	Other	18 (8.4 %)
	Unknown	32 (14.8 %)
HR status	ER+ and/or PR+	180 (83.3 %)
	ER– and PR–	36 (16.7 %)
Allocated treatment	TAC	109 (50.5 %)
	TAC + ZA	107 (49.5 %)
pCR breast and LN	Yes	25 (11.6 %)
	No	184 (85.2 %)
	Unknown	7 (3.2 %)
MP breast	1	33 (15.3 %)
	2	56 (25.9 %)
	3	41 (19.0 %)
	4	42 (19.4 %)
	5	35 (16.2 %)
	Unknown	9 (4.2 %)

*BMI* body mass index, *ER* estrogen receptor, *HR* hormone receptor, *LN* lymph nodes, *MP* Miller and Payne, *pCR* pathologic complete response, *PR* progesterone receptor, *TAC* docetaxel, doxorubicin, and cyclophosphamide, *ZA* zoledronic acid

### IGF-1R expression

**Fig. 2:**
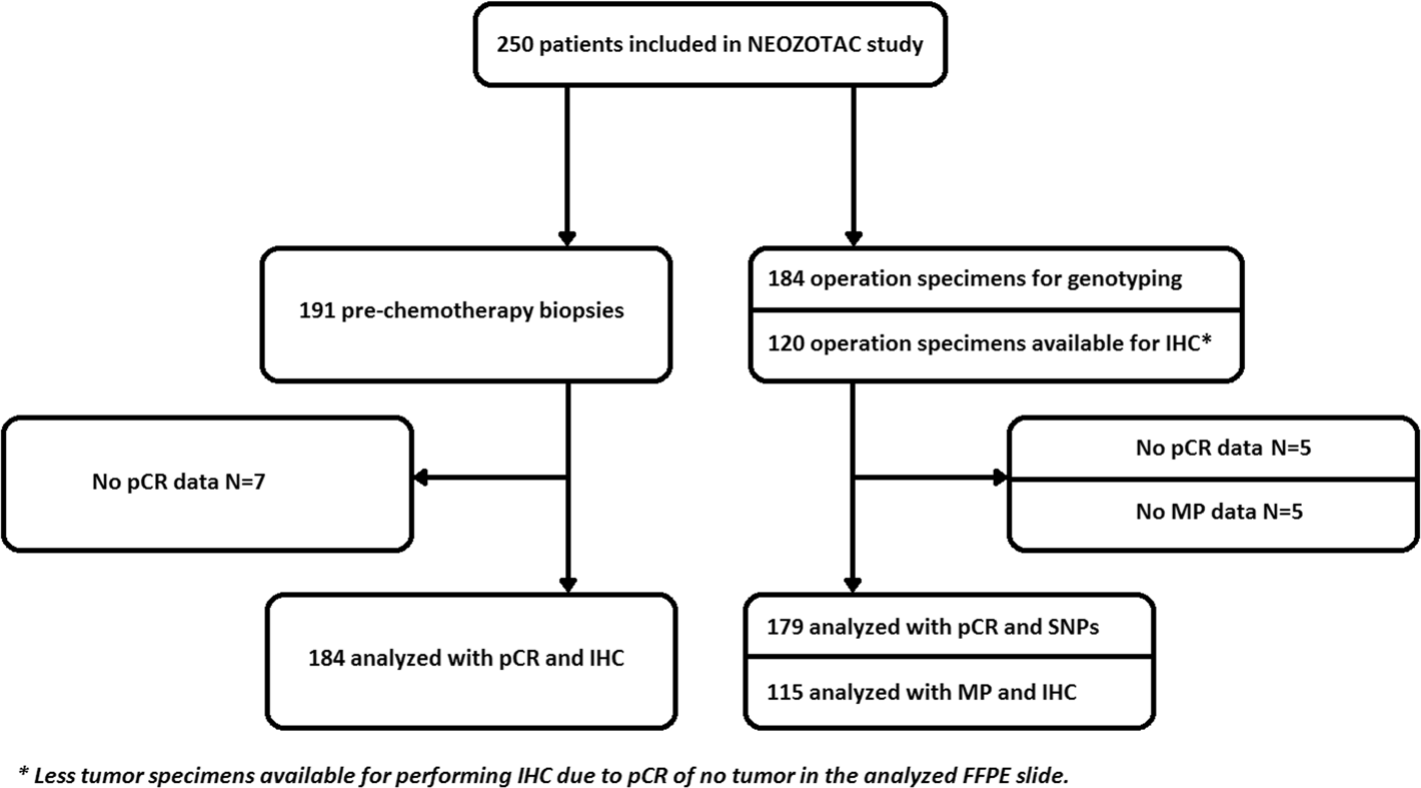
Consort diagram. *FFPE* formalin-fixed paraffin-embedded, *IHC* immunohistochemistry, *MP* Miller and Payne, *pCR* pathological complete response, *SNP* single nucleotide polymorphism. *Less tumor specimens available for performing IHC due to pCR of no tumor in the analyzed FFPE slide

FFPE breast tumor tissue from 216 patients was available for analyzing at least one condition (biopsy and/or operation specimen), while both samples were available for 106 cases. Data of available tissue are summarized in the consort diagram (Fig. 2). Representative tissue examples with different scoring values can be found in Fig. 1. High IGF-1R expression in the prechemotherapy biopsy was associated with ER expression (*P =* 0.001) and the progesterone receptor (PR) expression (*P =* 0.035). ER and/or PR-positive tumors showed positive IGF-1R on the membrane in 78.0 % of the cases, while triple-negative tumors showed positivity for IGF-1R in only 50.0 % of the cases.

**Fig. 3:**
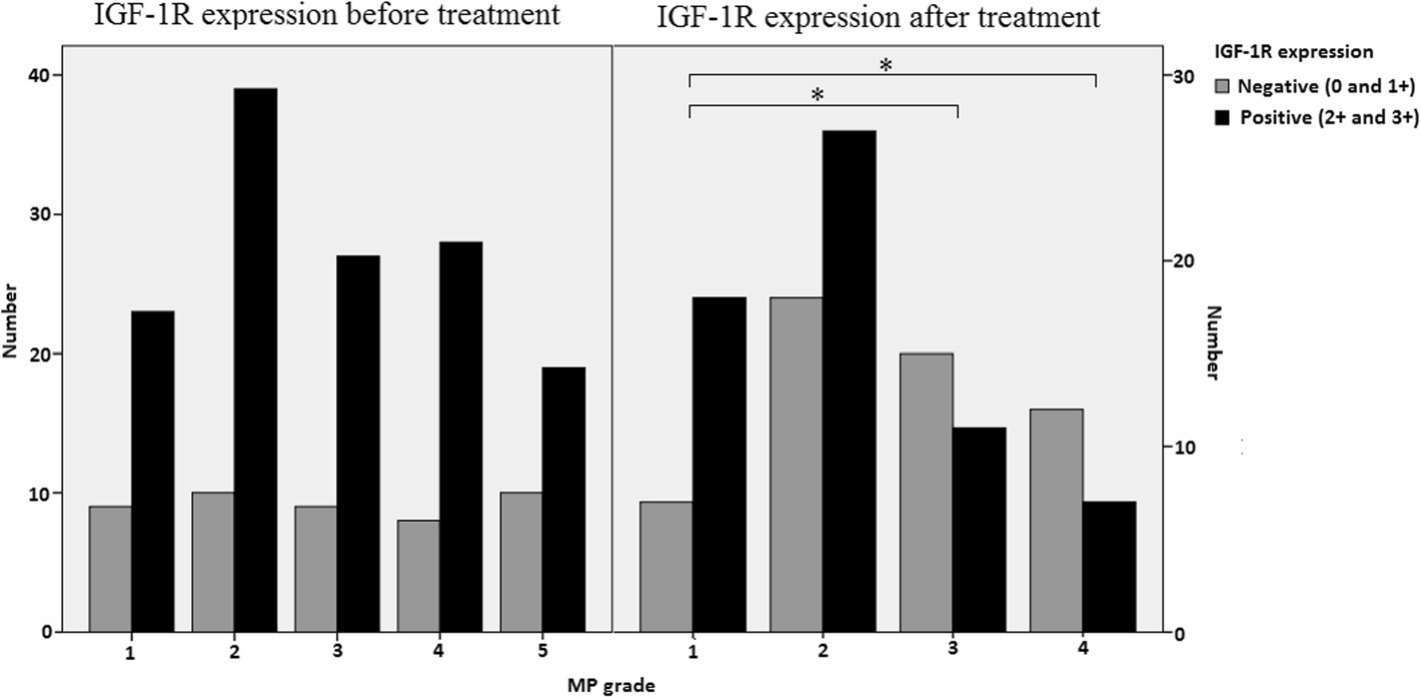
Membranous IGF-1R expression before and after treatment and the association with pathological response. *P <0.05. *IGF-1R* insulin-like growth factor 1 receptor, *MP* Miller and Payne

During chemotherapy, a significant subset (47.2 %), of tumors showed a decrease in IGF-1R expression while in a small subset of tumors the IGF-1R was upregulated (15.1 %). IGF-1R expression before treatment was not associated with pathological response (Fig. 3). However, the absence of IGF-1R expression (45 %) after treatment in the postchemotherapy operation specimens was associated with a better pathological response comparing ordinal MP classification response in univariate analysis (OR 2.60, 95 % CI 1.31–5.18, *P =* 0.006*)* (Fig. 3). This result remained significant in multivariate analysis when adjusting for HR status and clinical N stage (OR 2.64, 95 % CI 1.32–5.31, *P =* 0.006). With linear regression *P =* 0.008, indicating that the relationship between MP classification and IGF-1R expression is almost linear. Additionally, patients with a decrease in expression during treatment showed a better response to chemotherapy as well (OR 2.64, 95 % CI 1.17–5.98, *P =* 0.020 in multivariate analysis). Treatment with zoledronic acid had no influence on the IGF-1R expression in the operation specimen after treatment (*P =* 0.620) nor on diminished IGF-1R expression during treatment (*P =* 0.830) (data not shown).

### IGF-1R pathway SNPs

**Fig. 4:**
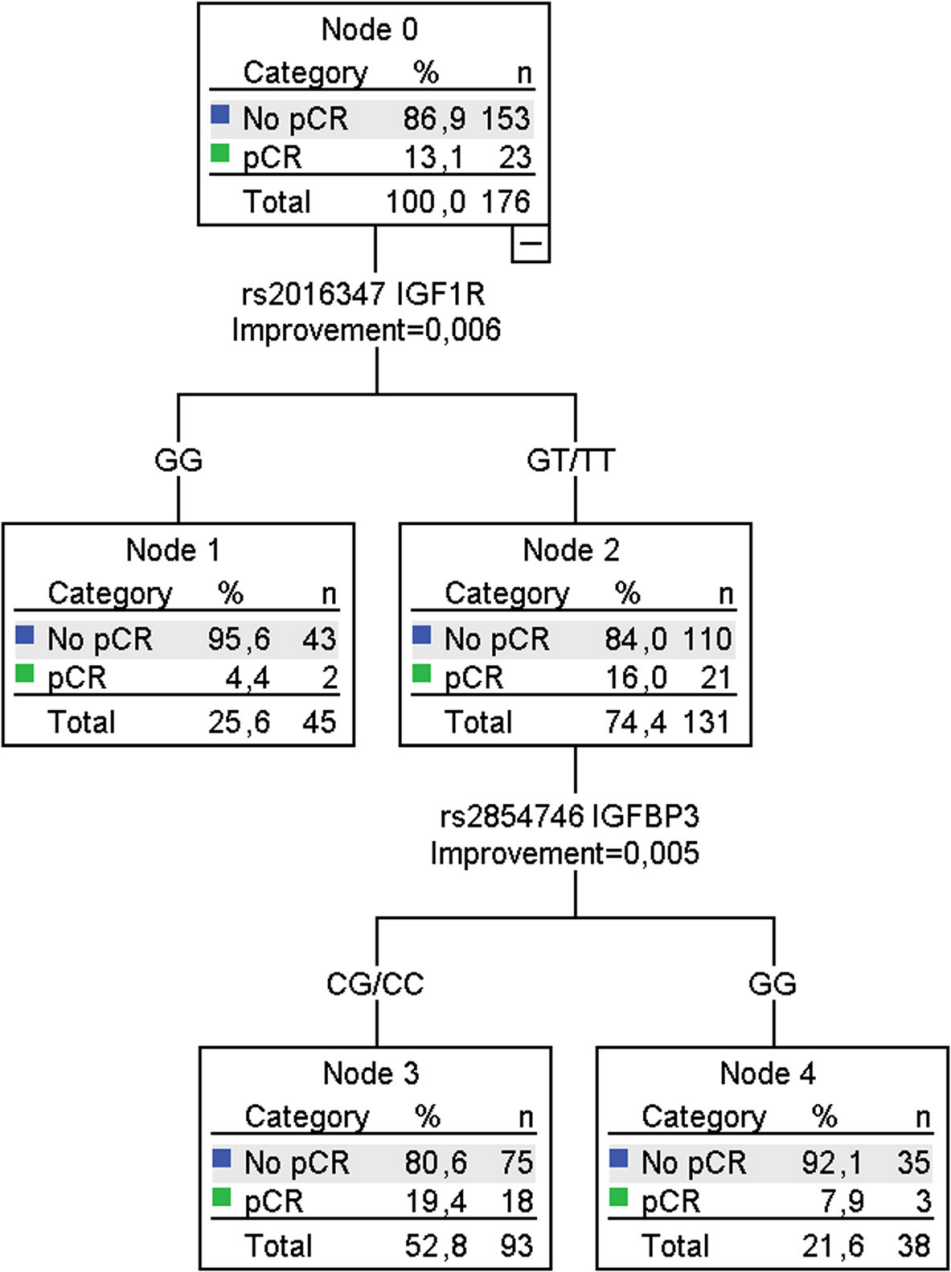
CART analyses of pCR in BC patients treated with TAC chemotherapy. The tree is divided by the SNPs to predict pCR, which has a significant prediction score (AUC 0.613 95 % CI 0.518–0.707, *P* = 0.040). *IGFBP3* insulin-like growth factor binding protein 3, *IGF1R* insulin-like growth factor 1 receptor, *pCR* pathological complete response

FFPE tissue samples from 184 (74 %) of 250 patients were available for analysis of IGF-1 pathway polymorphisms (preferentially tumor-negative tissue, see Methods). Data of available tissue are summarized in the consort diagram (Fig. 2). Of the eight genotyped SNPs, two significantly deviated from the Hardy–Weinberg equilibrium (rs2946834 and rs1520220). After correction for multiple testing, rs2946834 still significantly deviated from the Hardy–Weinberg equilibrium and was therefore excluded from the analysis. The genotype frequencies of rs1520220 did not differ from those observed in a publicly available database of European subjects (e.g., from the HapMap project) [28]. All eight SNPs had a call rate above 85 %, which is shown in Table 3. Clinical T stage, clinical N stage, and HR status were associated with pCR, wherefore was adjusted in multivariate analyses (Table 4). The variant T allele of 3129G > T in *IGF1R* (rs2016347) was associated with pCR in multivariate analysis (4.4 % for GG vs. 16.7 % GT/TT, *P =* 0.032) and the variant C allele of rs2854746 in *IGFBP3* tended to be associated with pCR in multivariate analysis (7.3 % for GG vs. 18.1 % GC/CC, *P =* 0.058). The global *P* value used for multiple testing correction for all eight SNPs together was *P* = 0.0095 for the dominant model (global score test). The CART derived from these SNPs is shown in Fig. 4. The corresponding AUC was 0.613 (95 % CI 0.518–0.707, *P =* 0.040).

**Table 3:** Distribution of genotypes of the investigated SNPs

SNP	Allele	*N* = 184 (%)	HWE χ^2^	*P* value	Call rate (%)
rs10735380	AA	110 (54.3)	2.1	0.144	94
*IGF1*	AG	68 (37.0)			
	GG	5 (2.7)			
	NE	11 (6.0)			
rs1520220	CC	115 (62.5)	4.4	0.040^a^	94
*IGF1*	CG	46 k			
	GG	11 (6.0)			
	NE	12 (6.5)			
rs6220	AA	91 (49.5)	3.3	0.068	89
*IGF1*	AG	56 (30.4)			
	GG	17 (9.2)			
	NE	20 (10.9)			
rs2946834^b^	GG	82 (44.6)	10.1	0.001^a^	88
*IGF1*	GA	53 (28.8)			
	AA	26 (14.1)			
	NE	23 (12.5)			
rs2270628	CC	105 (57.1)	2.8	0.096	87
*IGFBP3*	CT	45 (24.5)			
	TT	10 (5.4)			
	NE	24 (13.0)			
rs2854746	GG	59 (32.1)	1.9	0.170	90
*IGFBP3*	GC	72 (39.1)			
	CC	34 (18.5)			
	NE	19 (10.3)			
rs4320932	TT	111 (60.3)	0.04	0.843	96
*IGF2*	TC	57 (31)			
	CC	8 (4.3)			
	NE	8 (4.3)			
rs2016347	GG	48 (26.1)	1.8	0.185	96
*IGF1R*	GT	96 (52.2)			
	TT	32 (17.4)			
	NE	8 (4.3)			

^a^Not in HWE

^b^SNP excluded from analyses because the SNP is significantly deviated from HWE after Bonferroni correction

*HWE* Hardy–Weinberg equilibrium, *IGF* insulin-like growth factor, *IGFBP3* insulin-like growth factor binding protein 3, *IGF1R* insulin-like growth factor-1 receptor, *NE* Not evaluable (despite attempt to genotype), *SNP* single nucleotide polymorphism

**Table 4:** Associations between tumor and patient characteristics, SNPs, and pCR in breast and lymph nodes

				Univariate analysis			Multivariate analysis		
Parameter		*N*	% pCR	OR	95 % CI	*P* value	OR	95 % CI	*P* value
Clinical T stage	cT1/cT2	106	17.9	1	Reference		1	Reference	
	cT3/T4	73	6.8	0.34	0.12–0.95	0.039	0.49	0.16–1.50	0.209
Clinical N stage	cN0	*84*	21.4	1	Reference		1	Reference	
	cN+	*95*	6.3	0.25	0.09–0.66	0.005	0.19	0.06–0.58	0.003
HR status	ER+ and/or PR+	151	8.6	1	Reference		1	Reference	
	Triple negative	28	39.3	6.87	2.66–17.7	0.00007	9.35	3.09–28.3	0.00008
Allocated treatment	TAC + ZA	87	14.9	1	Reference	0.559			
	TAC only	92	12.0	0.77	0.33–1.83				
Age				0.96	0.89–1.09	0.186			
BMI				0.97	0.88–1.08	0.581			
rs10735380	AA	97	13.4	1	Reference				
*IGF1*	AG	66	13.6	1.02	0.41–2.55	0.966			
	GG	5	20.0	1.61	0.17–15.6	0.679			
rs1520220	CC	111	15.3	1	Reference				
*IGF1*	CG	45	13.3	0.85	0.31–2.32	0.752			
	GG	11	0.0	–	–	–			
rs6220	AA	88	11.4	1	Reference				
*IGF1*	AG	56	16.1	1.49	0.57–3.94	0.418			
	GG	17	17.6	1.67	0.41–6.48	0.475			
rs2270628	CC	101	11.9	1	Reference				
*IGFBP3*	CT	45	17.8	1.60	0.61–4.24	0.342			
	TT	9	0.0	–	–	–			
rs2854746	GG	55	7.3	1	Reference		1	Reference	
*IGFBP3*	GC	72	16.7	2.55	0.78–8.40	0.124	3.06	0.82–11.4	0.097
	CC	33	21.2	3.43	0.92–12.8	0.066	4.02	0.92–17.6	0.065
	GG	55	7.3	1	Reference		1	Reference	
	GC/CC	105	18.1	2.82	0.91–8.74	0.073	3.35	0.96–11.7	0.058
rs4320932	TT	106	15.1	1	Reference				
*IGF2*	TC	57	12.3	0.79	0.30–2.04	0.623			
	CC	8	12.5	0.80	0.09–6.98	0.843			
rs2016347	GG	45	4.4	1	Reference		1	Reference	
*IGF1R*	GT	94	17.0	4.41	0.97–20.1	0.055	5.58	1.08–28.7	0.040
	TT	32	15.6	3.98	0.72–22.0	0.113	6.67	1.03–43.1	0.046
	GG	45	4.4	1	Reference		1	Reference	
	GT/TT	126	16.7	4.30	1.00–19.1	0.056	5.82	1.17–29.1	0.032

*BMI* body mass index, *CI* confidence interval, *ER* estrogen receptor, *HR* hormone receptor, *IGF* insulin-like growth factor 1, *IGFBP3* insulin-like growth factor binding protein 3, *IGF1R* insulin-like growth factor 1 receptor, *OR* odds ratio, *pCR* pathological complete response, *PR* progesterone receptor, *SNP* single nucleotide polymorphism, *TAC* docetaxel, doxorubicin, cyclophosphamide, *ZA* zoledronic acid

Moreover, the variant T allele of C > T in *IGFBP3* (rs2270628) was associated with a higher occurrence of grade III/IV side effects in univariate analysis (OR 2.20, 95 % CI 1.04–4.67, *P =* 0.039) and multivariate analysis (18.1 % for CC vs. 32.7 % CT/TT, OR 2.30, 95 % CI 1.06–4.98, *P =* 0.034) (data not shown). The multivariate analysis was adjusted for body mass index, as it was significantly associated with grade III/IV side effects.

### Genotype–phenotype associations

rs2016347 in *IGF1R* was not associated with IGF-1R expression before chemotherapy (78.3 % for GG vs. 65.9 % GT/TT, *P =* 0.115) or after chemotherapy (50.0 % for GG vs. 67.7 % GT/TT, *P =* 0.099).

## Discussion

This translational study showed that IGF-1R expression changed in most of the tumors during treatment in stage II/III HER2-negative BC patients treated with neoadjuvant TAC chemotherapy and that absent or diminished expression after treatment was associated with a better pathological response according to MP classification. Additionally, we found that the variant T allele of 3129G > T in *IGF1R* (rs2016347) was significantly associated with a better pathological response according to MP classification after neoadjuvant chemotherapy.

Changes of IGF-1R expression of the tumor during chemotherapy have been described previously [18, 31]. Our study confirms these results in a larger and more homogeneous patient cohort. Moreover, in the current trial a greater part of the tumors showed a decline in IGF-1R expression (47.2 %) compared with the prior described 14.0 %. This might be explained by the difference in chemotherapy regimens used as well as the absence of HER2 expression in our cohort, as HER2-positive tumors show less IGF-1R expression [10, 11]. The decline of IGF-1R expression in the tumor during TAC treatment observed in our study might reflect chemotherapy efficacy, as patients with a decline in IGF-1R expression showed a significantly better pathological response than tumors with no change or an increase in expression. In keeping with this inference, downregulation of IGF-1R during chemotherapy treatment is associated with prolonged survival [18]. Bhargava et al. [10] showed that low IGF-1R expression before treatment was associated with a better response to neoadjuvant chemotherapy in ER-positive tumors, but not in triple-negative tumors. We could not reproduce this association, but this could be explained by the difference in cohort (e.g., differences in HER2 status and chemotherapy regimen).

In our exploratory analysis of IGF-1 pathway polymorphisms, the variant T allele of 3129G > T in *IGF1R* (rs2016347) was associated with a better pathological response according to MP classification after neoadjuvant chemotherapy. This is in accordance with studies that associated 3129G > T in *IGF1R* (rs2016347) with cancer prognosis and treatment outcome [22, 32, 33]. Winder et al. [22] found that the T allele was associated with a better overall survival in colorectal cancer patients treated with cetuximab [33] and a better overall survival in ER-positive BC patients treated with tamoxifen. rs2016347 is localized in the 3′-untranslated region of the *IGF1R* gene, functioning as a microRNA binding site [28]. Because microRNA binding sites are important for mRNA translation and degradation, the variant T allele of rs2016347 might disturb binding to this microRNA site [34]. Although the precise functional effect of *IGF1R* rs2016347 is unknown, it would be a plausible explanation that the T allele of rs2016347 may reduce IGF-1R expression. However, in our study rs2016347 in *IGF1R* was not associated with IGF-1R expression.

The variant T allele of C > T in *IGFBP3* (rs2270628) was associated with the occurrence of grade III/IV side effects. Although the mechanism is unclear, several studies have shown that the variant T allele of rs2270628 is associated with decreased serum IGF-BP3 levels [35, 36]. IGF-1 activity depends on binding with IGF-BP3 [12, 13], so it may be that higher activity of IGF-1 due to lower levels of IGF-BP3 causes a higher incidence of toxicity of chemotherapy in our study [6].

Our study has some limitations. Using our approach, we could not investigate the best responders (MP5) after chemotherapy because inherently no tumor tissue was left to measure IGF-1R in the operation specimen. Moreover, the response of the lymph nodes is not evaluated in the MP grading system because it focuses only on the primary tumor. Although, the survival of patients with a partial response is affected by residual lymph node status [37]. Additionally, the number of evaluable triple-negative tumors was too small to evaluate for differences in response associated with IGF-1R between HR-positive tumors and triple-negative tumors. Our sample size for the explorative genotype–phenotype optional side study was small and this was probably the reason why we could not reproduce the associations between the serum IGF-1 and IGF-BP3 levels and SNPs. However, the results of our study provide further evidence for the importance of patient selection for (co)treatment with an IGF-1 inhibitor. Until now no convincing benefit of IGF-I pathway inhibitors was found in clinical studies in BC [38–40]. These studies lacked patient selection based on IGF-1 pathway activity. It may be hypothesized that patients with a diminished IGF-1R after chemotherapy will not benefit from an IGF-1R inhibitor, while a patient with upregulated IGF-1R might benefit.

## Conclusions

IGF-1R expression in the tumor changed during chemotherapy and absent or diminished expression of IGF-1R after treatment was associated with a better pathological response. rs2016347 in *IGF1R* was associated with pCR after TAC chemotherapy. These observations may help to predict the efficacy of TAC chemotherapy and to select patients who might benefit from (co)treatment with an IGF-1 pathway inhibitor.

## Abbreviations

AUC: Area under the curve
BC: Breast cancer
BSA: Bovine serum albumin
CART: Classification and regression tree
CI: Confidence interval
CTCAE: Common Terminology Criteria for Adverse Events
ER: Estrogen receptor
FFPE: Formalin-fixed paraffin-embedded
HR: Hormone receptor
IGF: Insulin-like growth factor
IGF-1R: Insulin-like growth factor 1 receptor
IGF-BP: Insulin-like growth factor binding protein
IHC: Immunohistochemistry
MAF: Minor allele frequency
MP: Miller and Payne
OR: Odds ratio
PBS: Phosphate-buffered saline
pCR: Pathological complete response
PR: Progesterone receptor
SNP: Single nucleotide polymorphism
TAC: Docetaxel, doxorubicin, and cyclophosphamide
